# Impact of Fear of COVID-19, Depression, Anxiety and Stress on Temporomandibular Disorders in Peruvian Dental Students in the Post-Pandemic Period: A Multivariable Regression Analysis

**DOI:** 10.3390/jcm13154410

**Published:** 2024-07-28

**Authors:** Manuel Castro-Mena, Jose Huamani-Echaccaya, Enrique Yarasca-Berrocal, Marysela Ladera-Castañeda, Miriam Castro-Rojas, Rosa Aroste-Andía, Cinthia Hernández-Vergara, Luis Cervantes-Ganoza, César Cayo-Rojas

**Affiliations:** 1 School of Stomatology, Universidad Privada San Juan Bautista, Lima 15067, Peru; 2 School of Stomatology, Universidad Privada San Juan Bautista, Ica 11004, Peru; 3 Postgraduate School, Universidad Nacional Federico Villarreal, Lima 15001, Peru; 4 Faculty of Stomatology, Universidad Inca Garcilaso de la Vega, Lima 15084, Peru

**Keywords:** depression, anxiety, stress, COVID-19, dental students, fear, temporomandibular disorders

## Abstract

**Background**: Dentists, who frequently encounter potentially infected patients, have experienced significant changes worldwide due to the COVID-19 pandemic. The aim of this study was to evaluate the impact of the fear of COVID-19, depression, anxiety and stress on the presence of temporomandibular disorders (TMD), taking into account possible confounding variables, in Peruvian dental students during the post-pandemic period. **Methods**: This analytical cross-sectional study assessed 607 Peruvian dental students from two regions of Peru. The study utilized the Depression Anxiety Stress Scales-21 (DASS-21), the Fear of COVID-19 Scale (FCV-19S) and the Short Form of the Fonseca Anamnestic Index (SFAI). Possible confounding variables were sex, age, year of study, marital status, place of origin, area of residence, history of mental illness and living with people vulnerable to COVID-19. For the multivariable analysis, we utilized a Poisson regression model with an adjusted robust variance. The significance level was set at *p* < 0.05. **Results**: The rates of depression, anxiety, stress, fear of COVID-19 and temporomandibular disorders were 47.0%, 50.4%, 35.9%, 30.6% and 54.2%, respectively. Moreover, the study revealed that students with depression and anxiety were 38% and 75% more likely to have temporomandibular disorders compared to those without depression (APR = 1.38, 95% CI: 1.15–1.66) and anxiety (APR = 1.75, 95% CI: 1.44–2.13), respectively. Similarly, the likelihood of presenting temporomandibular disorders was 55% higher in women than in men (APR = 1.55, 95% CI: 1.28–1.87). Furthermore, we found that stress and fear of COVID-19 did not determine the development of temporomandibular disorders (*p* > 0.05). **Conclusions:** Almost half of the dental students experienced depression, anxiety and TMD in the post-pandemic period. In addition, depression and anxiety were influential factors in the occurrence of TMDs, with the female gender being a risk factor. However, factors such as fear of COVID-19, stress, age, year of study, marital status, place of origin, area of residence, history of mental illness or living with people vulnerable to COVID-19 were not significant.

## 1. Introduction

In December 2019, the SARS-CoV-2 virus originated in Wuhan, China, causing severe acute pneumonia in many infected patients and leading to a deadly pandemic [1]. This virus has caused numerous deaths worldwide and has also resulted in economic and social problems [2]. The COVID-19 pandemic has significantly affected the daily lives of adults and children, forcing people to adapt to new safety measures such as wearing masks and social distancing [3]. This has led to increased concern for personal health and well-being, which in turn has impacted mental health [4,5].

Although the pandemic had a significant psychological impact on the general population, it also presented significant challenges for healthcare professionals. At the start of the pandemic, shortages of medicines, personal protective equipment, specialized environments and equipment for intensive care units were common, resulting in physical and emotional strain on many healthcare workers [6,7]. Despite the challenging circumstances during the early stages of the pandemic, many healthcare workers, including dentists, persisted in providing their services to the population. This was despite the high risk of contagion due to direct contact with saliva, the primary biological agent of COVID-19 infection [8,9]. Therefore, it is natural for dental students and professionals to experience fear of this disease, as it has claimed thousands of lives in the healthcare sector [10,11,12,13].

The aforementioned reasons led to a temporary suspension of dental practices at the beginning of the pandemic, as dentistry was identified as one of the areas with the highest risk of contagion [14,15,16,17]. However, by 2023, dental professionals, particularly those with vulnerable conditions or those living with family members with pre-existing chronic diseases, were providing care services normally despite the potential risk [15]. Recent research indicates that this situation may impact dental students, as their professional training mandates them to interact with potentially infected patients during their clinical practices or hospital internships [18,19,20,21]. Despite receiving multiple doses of the COVID-19 vaccine in 2023, students may still fear the disease due to the persistently high rates of infection and death. In March of that year, Peru reported up to 385 deaths per day [22].

During the COVID-19 pandemic in Brazil and Turkey, reports indicated that certain behavioral disorders could lead to bruxism, potentially leading to temporomandibular disorders [23,24,25]. Given the multifactorial etiology of TMD, which involves alterations of the stomatognathic system in relation to the temporomandibular joint and its musculoskeletal structures, psychological disorders could potentially contribute to its cause. Furthermore, environmental, biological, psychological, biomechanical, and/or neuromuscular factors may affect the etiology of TMDs [23]. Clinical evidence has subsequently demonstrated the relationship between TMDs and psychological disorders [26].

As of October 2023, only one study has been reported in the Latin American region, which was conducted in Brazil. Therefore, it is crucial to continue this line of research in other countries worldwide [24]. This study is significant because it is necessary to understand the mental health status of students in the post-pandemic context of COVID-19. Additionally, we are analyzing a possible associated factor, such as fear of COVID-19, which may influence the development of TMDs [27,28]. The short version of the Fonseca Anamnestic Index (SFAI) has demonstrated very good results in predicting TMD compared to the Diagnostic Criteria for Temporomandibular Disorder (DC/TMD) protocol, which is the gold standard in TMD prediction [29,30,31]. The SFAI is a 10-item questionnaire-type instrument that assesses the frequency of pain, psychological distress, the limitation of jaw function and parafunctional behaviors associated with TMDs. This instrument has the advantage of allowing for the assessment of large populations in a shorter time, making it suitable for epidemiological evaluations as well as clinical screening methods in daily dental practice [31].

The early detection of these pathologies could spur university authorities to develop preventive strategies with appropriate professional assistance. This could help to reduce the psychological impact on dental students, as well as the impact on oral health.

The aim of this study was to evaluate the impact of the fear of COVID-19, depression, anxiety and stress on the presence of temporomandibular disorders, taking into account possible confounding variables in Peruvian dental students during the post-pandemic period. The study’s null hypothesis was that fear of COVID-19, depression, anxiety and stress had no influence on the presence of temporomandibular disorders in these students.

## 2. Materials and Methods

### 2.1. Study Design

The School of Stomatology of the Universidad Privada San Juan Bautista (UPSJB), located in Lima, Peru, with a branch in Ica, conducted this cross-sectional, analytical and prospective study between 24 April and 30 June 2023. The study adhered to the Strengthening the Reporting of Observational Studies in Epidemiology (STROBE) guidelines for observational studies [32].

### 2.2. Population and Selection of Participants

**Figure 1:**
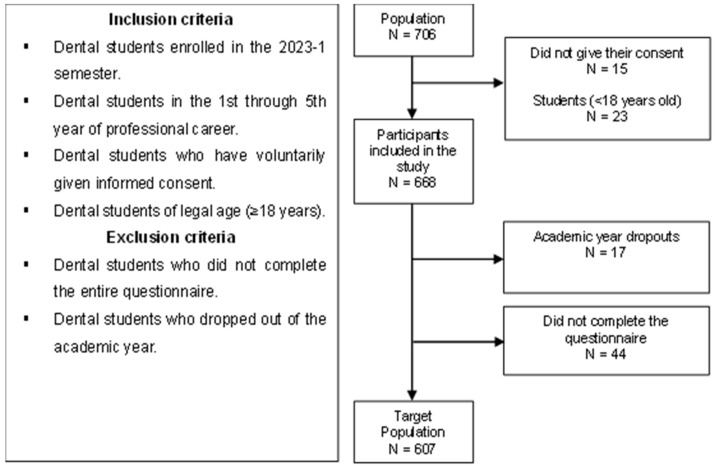
Flowchart on participant selection.

The population consisted of 706 dental students from UPSJB (311 from the Peruvian province of Ica and 395 from Lima, the capital city). The total number of participants consisted of 162 first-year students, 148 second-year students, 159 third-year students, 112 fourth-year students and 125 fifth-year students. To calculate the sample size, we used a formula in the Epidat 4.2 statistical package to estimate a proportion with a finite population, considering a confidence level of 95%, an expected proportion of 50% (to obtain the largest sample size), and an estimation error of 5%. The calculated sample size was n = 249. This led to the inclusion of the entire study population, which included 122 first-year students, 135 second-year students, 126 third-year students, 103 fourth-year students and 121 fifth-year students [Figure 1]. 

### 2.3. Variables

Temporomandibular disorders [28] were considered a dependent variable, and depression, anxiety, stress and fear of COVID-19 (the questions are designed to elicit information about the fear experienced at the time of the survey) [33] were considered independent variables. Additionally, we considered sex, age, year of study, marital status, place of origin, area of residence, history of mental illness and living with vulnerable individuals (older adults with co-morbidities) as potential confounding variables [33,34].

### 2.4. Instruments

We delivered a heteroadministered survey using an in-person questionnaire as part of the data collection process. We identified psychological symptomatology using the DASS-21 scale. The questionnaire contained twenty-one items, categorized into three categories: stress, anxiety and depression. We composed seven randomly distributed questions for each dimension: Each survey item provided four ordinal responses on a Likert scale: “never” (0 points), “sometimes” (1 point), “often” (2 points) and “always” (3 points). We summed the scores for each component to diagnose stress, anxiety and depression. We identified anxiety in participants who scored between 4 and 21 points, depression in those who scored between 5 and 21 points and stress in those who scored between 8 and 21 points [35,36]. The Cronbach’s alpha (α) values for depression, anxiety and stress were 0.884 (95% CI: 0.870–0.898), 0.843 (95% CI: 0.823–0.861) and 0.863 (95% CI: 0.846–0.879), respectively. These values were considered adequate. 

The Fear of COVID-19 Scale questionnaire consists of seven items rated on a Likert scale ranging from 1 (strongly disagree) to 5 (strongly agree). A score of 17 to 35 indicates a diagnosis of fear of COVID-19 [37]. The questionnaire’s reliability analysis, according to Cronbach’s alpha (α), was 0.886 (95% CI: 0.871–0.899), which is considered acceptable.

We used the Fonseca Short Form Anamnestic Index (SFAI) to detect temporomandibular disorders (TMD). The SFAI comprises 10 questions with three alternatives: ‘yes’ (10 points), ‘sometimes’ (5 points) and ‘no’ (0 points). We calculated the final score by adding the points for each item, and a score of 20 to 100 points was considered indicative of TMD [31]. The instrument’s reliability analysis, as measured by Cronbach’s alpha (α), was 0.742 (95% CI: 0.710–0.771), which is considered acceptable.

### 2.5. Procedure

The students were provided with the questionnaires in person by the principal investigator (MCM), who also elucidated the rationale behind the study. It should be noted that this individual was not a professor of the students who were surveyed. The first page of the questionnaire included an informed consent form with the contact information of the Institutional Research Ethics Committee, as well as the full name, email address and telephone number of the principal investigator. If the students provided their consent, they proceeded to the next page, which included instructions for completing the questionnaire. Participants could decline the invitation or not complete the questionnaire. The principal investigator was the only one with access to the participants’ information. To maintain data confidentiality, the information was stored on a portable digital device with a password. Only one complete response per student was accepted. To avoid the duplication of responses, the participants were instructed to write their initials in the header: first name, surname and age (e.g., MCM24). Subsequently, to anonymize the data, the initials were replaced by sequential numeric characters after randomizing the order of the participants in the database. No incentives were provided to guests for their participation.

### 2.6. Data Analysis

We performed the statistical analysis with the SPSS (IBM Corp., Armonk, NY, USA) v.28.0 program and imported the data from a Microsoft Excel 2019 spreadsheet. For the descriptive analysis of qualitative variables, we used absolute and relative frequencies. We used measures of central tendency, such as the mean and median, as well as the standard deviation, as a measure of dispersion for the quantitative variable “age”. To determine the association between sociodemographic variables and depression, anxiety, stress, fear of COVID-19 and the presence of temporomandibular disorders, we used Pearson’s chi-square test. This test allowed us to determine whether the distribution of the observed response is random or significantly associated with any of the variables [38]. For multivariate analysis, we used a Poisson multiple regression model with robust variance and an adjusted prevalence ratio (APR). This helped us determine how fear of COVID-19, depression, anxiety and stress affected the number of people with TMD while also accounting for any possible confounding variables. The significance level was set at *p* < 0.05 for all statistics.

### 2.7. Ethical Aspects

The present research adhered to the bioethical principles outlined in the Declaration of Helsinki, including respect, freedom, non-maleficence and confidentiality [39]. The UPSJB Institutional Research Ethics Committee granted approval with resolution No. 0518-2023-CIEI-UPSJB, dated 17 April 2023. Additionally, the first page requested voluntary, informed consent from the students.

## 3. Results

The participants’ mean age was 22.6 ± 5.2 years, with 52.1% being 21 years old or younger and 66.1% being female. Student participation varied from 17.0% to 22.2% across academic years. The majority of participants were single (83.2%), and 52.1% were from the capital city. Additionally, 89.8% of respondents lived in urban areas. However, only 4.1% of the total had a history of mental illness, and 20.9% of the students lived with people vulnerable to COVID-19 [Table 1].

**Figure 2:**
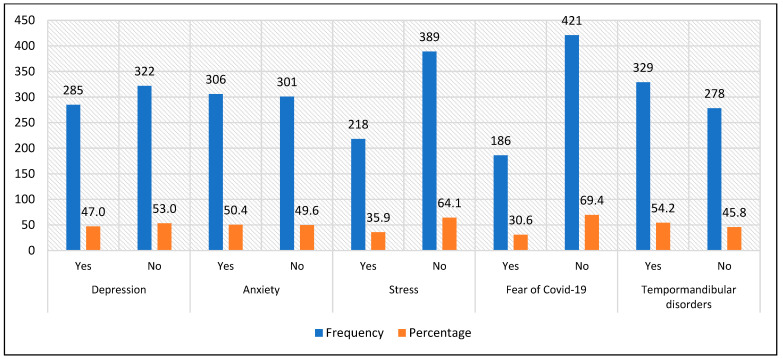
Absolute and relative frequency of depression, anxiety, stress, fear of COVID-19 and temporomandibular disorders in dental students.

The values for depression, anxiety, stress, fear of COVID-19 and the presence of temporomandibular disorders in the surveyed dental students were 47.0% (95% CI: 43.0%–50.9%), 50.4% (95% CI: 46.4%–54.4%), 35.9% (95% CI: 32.1%–39.7%), 30.6% (95% CI: 27.0%–34.3%) and 54.2% (95% CI: 50.2%–58.2%), respectively [Figure 2].

Females have a significantly higher rate of depression, anxiety, stress, fear of COVID-19 and temporomandibular disorders compared to males (*p* = 0.029, *p* = 0.004, *p* = 0.001, *p* = 0.024 and *p* < 0.001, respectively). Additionally, students aged 21 years or younger have a significantly higher rate of depression and anxiety compared to those older than 21 years (*p* = 0.007 and *p* < 0.001, respectively). The fear of COVID-19 was significantly associated with the year of study (*p* = 0.018). Additionally, single individuals had a significantly higher rate of anxiety compared to married individuals (*p* = 0.041). Furthermore, those from the province had a significantly higher rate of depression, anxiety, fear of COVID-19 and temporomandibular disorders compared to those from the capital (*p* < 0.001, *p* = 0.001, *p* = 0.024 and *p* = 0.045, respectively). Finally, it was observed that individuals with a history of mental illness had a significantly higher rate of depression, anxiety and stress compared to those without (*p* < 0.001, *p* < 0.001 and *p* < 0.001, respectively) [Table 2].

The multivariable Poisson regression model with robust variance and temporomandibular disorders (yes = 1 and no = 0) as the dependent variables showed that students who are depressed are 38% more likely to have temporomandibular disorders than students who are not depressed (APR = 1.38, 95% CI: 1.15–1.66). Students with anxiety had a 75% higher likelihood of having temporomandibular disorders compared to those without anxiety (APR = 1.75, 95% CI: 1.44–2.13). Women were 55% more likely to present temporomandibular disorders than men (APR = 1.55, 95% CI: 1.28–1.87). Stress and fear of COVID-19 did not significantly contribute to the development of temporomandibular disorders (*p* > 0.05). Similarly, we found no significant influence from age, year of study, marital status, origin, history of mental illness or living with people vulnerable to COVID-19 (*p* > 0.05) [Table 3].

## 4. Discussion

The COVID-19 pandemic has significantly impacted various aspects of our society, including work, the economy and education [33,40]. Despite the post-pandemic context, concerns remain due to the persistence of complications that can lead to mortality [41]. Due to the high risk of COVID-19 infection from frequent contact with saliva, health science students, particularly those in dentistry, may experience persistent levels of anxiety and depression [23]. Additionally, this situation may contribute to the development of other conditions, such as temporomandibular disorders, due to psychological distress [42,43]. Therefore, the aim of this study was to evaluate the impact of the fear of COVID-19, depression, anxiety and stress on the presence of TMD, taking into account possible confounding variables in Peruvian dental students during the post-pandemic period. We partially rejected the null hypothesis, finding an association between depression, anxiety and TMDs but not between stress and fear of COVID-19 and TMDs.

The results obtained are consistent with those studied by Gas et al. [25], who found that the rate of depression, anxiety, stress and temporomandibular disorders was significantly higher in females than in males. The higher frequency of mental illness and living with vulnerable individuals among female students in the present study could potentially contribute to the development of these symptoms. However, the results obtained by Fauzi et al. [44] differ from those of the present study, as they reported no significant difference in the rate of depression, anxiety and stress between male and female students. This discrepancy may be due to the small sample size of male students in the study by Fauzi et al., which may not have been sufficient to identify differences. For instance, hormonal changes may cause depression, anxiety and stress in women, which can affect their mental health. Contrarily, men typically experience more cognitive effects than emotional ones [45].

A noteworthy finding of this study is that, according to bivariate analysis, students in the provinces showed a significantly higher rate of depression, anxiety and fear of COVID-19 compared to students in the capital. The pandemic’s economic hardship may make it difficult for students in some provinces to access basic health resources for psychological support and to afford private university education. This may contribute to the development of psychological disorders. However, Wathelet et al. [46] reported that students far from the capital were less likely to develop depressive and anxious symptoms due to the reassurance of living in less populated areas with a lower risk of becoming infected with COVID-19 or other diseases. The timing of Wathelet et al.’s study during the initial wave of contagion in 2020, when social distancing was widely associated with well-being, may explain the discrepancy between the results. Our study took place in 2023, a time when COVID-19 had already resulted in numerous fatalities, particularly in provinces with inadequate drug supplies and hospital facilities for critical care. In addition, the vaccination process in the provinces was slow [47]. 

This study found that students with depression and anxiety were 38% and 75% more likely to present TMD, respectively, under a multivariable regression model compared to those without these conditions. The study also found that stress and fear of COVID-19 were not determinants for developing TMD. These results are partially consistent with those of Namvar et al. [48], who conducted a study in Iran and found that depression, anxiety and stress significantly influence the development of TMDs. Furthermore, they concluded that stress was the most influential factor in this disorder. One potential explanation for this discrepancy is that the research in Iran was conducted in 2021, during the height of the global pandemic caused by COVID-19. It is therefore reasonable to conclude that the Iranian population experienced a greater number of stressors, including a lack of vaccines, a high daily mortality rate, mandatory social isolation and infodemia (the considerable amount of data, comprising both verifiable information and unsubstantiated claims, making it difficult to determine a reliable source of guidance) [49,50]. In contrast, our research was conducted two years later, when more accredited and reliable information was available. Dental care protocols had been developed to mitigate the wave of coronavirus infections, and many individuals had already received three or four doses of the COVID-19 vaccine [51]. However, according to studies by Bangasser et al. and Gao et al. [52,53], women are more likely to experience anxiety and depression due to neuroticism, a personality trait characterized by a negative emotional response to potential threats, frustrations or losses. Anxiety and depression directly correlate with this trait [54,55]. Given the direct association between anxiety, depression and TMD in this study, it is likely that women are 55% more likely to develop TMD than men in a context where life is still at risk due to COVID-19. 

As dental students advance in their professional careers, it is inevitable that they will come into contact with patients potentially infected with COVID-19, which could transmit a certain degree of emotional distress, even more so if these students live with vulnerable people [56,57]. It is crucial to evaluate and analyze the mental health status of dental students and the potential pathophysiological consequences of behavioral disorders in the stomatognathic system. Constant psychological support and interdisciplinary medical consultation, including with a dentist and/or otolaryngologist, are essential to prevent possible behavioral disorders that may cause temporomandibular dysfunction. Certain personality traits may hinder resilience and increase the likelihood of developing anxious or depressive symptoms. In turn, this can make the masticatory muscles in the temporomandibular area tense and hyperactive. It can also cause improper oral habits that hurt your face, cause chronic muscle tension and make the temporomandibular joints not work right [58,59]. 

A limitation of this study is that it only included dental students from a single university in the capital city, with a branch in one province. We implemented this strategy to account for academic demands within the same curriculum, given the reported impact of academic demands on students’ emotional states [60]. Another limitation was that each diagnosis was based on the student’s symptoms reported using questionnaires. While we acknowledge that these findings may not apply to the entire country, they can provide a valuable foundation for identifying and understanding students’ mental health status and implementing necessary measures to safeguard their psychological well-being. Furthermore, the cross-sectional design of this study precluded the assessment of the TMD’s longevity over time. 

Academic authorities should consider implementing mental health prevention measures or providing psychological support plans for their students. A beneficial starting point for these plans would be the preparation of mental health manuals or guides to address stress, depression, anxiety or other psychological problems [5]. The institution’s psychology department would be ideal for constant student monitoring. We recommend replicating this study in other Peruvian universities with different social realities to evaluate the potential relationship between fear of COVID-19, depression, anxiety and stress and the development of TMDs in students.

## 5. Conclusions

Almost half of the dental students experienced depression, anxiety and TMD in the post-pandemic period. In addition, depression and anxiety were influential factors in the occurrence of TMDs, with female sex being a risk factor. However, factors such as the fear of COVID-19, stress, age, year of study, marital status, place of origin, area of residence, history of mental illness or living with people vulnerable to COVID-19 were not significant.

## Figures and Tables

**Table 1:** Sociodemographic characteristics of dental students.

Variable	Category	Frequency	Percentage
**Sex**	Female	401	66.1
	Male	206	33.9
**Age group**	≤21 years	316	52.1
	>21 years	291	47.9
**Year of study**	1st year	122	20.1
	2nd year	135	22.2
	3rd year	126	20.8
	4th year	103	17.0
	5th year	121	19.9
**Marital status**	Single	505	83.2
	Married or cohabiting	102	16.8
**Place of origin**	Capital	316	52.1
	Province	291	47.9
**Area of residence**	Urban	545	89.8
	Rural	62	10.2
**History of mental illness**	Yes	25	4.1
	No	582	95.9
**Living with people vulnerable to COVID-19**	Yes	127	20.9
	No	480	79.1
**Age**	**Mean**	**Median**	**SD**
	22.6	21.0	5.2

SD: Standard Deviation.

**Table 2:** Sociodemographic variables of dental students associated with the rate of depression, anxiety, stress, fear of COVID-19 and the presence of temporomandibular disorders.

Variable	Category	Depression			Anxiety			Stress			Fear of COVID-19			Temporomandibular Disorders		
		Yes	No	*p* *	Yes	No	*p* *	Yes	No	*p* *	Yes	No	*p* *	Yes	No	*p* *
		f (%)	f (%)		f (%)	f (%)		f (%)	f (%)		f (%)	f (%)		f (%)	f (%)	
**Sex**	Female	201 (50.1)	200 (49.9)	0.029 *	219 (54.6)	182 (45.4)	0.004 *	162 (40.4)	239 (59.6)	0.001 *	135 (33.7)	266 (66.3)	0.024 *	253 (63.1)	148 (36.9)	<0.001 *
	Male	84 (40.8)	122 (59.2)		87 (42.2)	119 (57.8)		56 (27.2)	150 (72.8)		51 (24.8)	155 (75.2)		76 (36.9)	130 (63.1)	
**Age group**	≤21 years	165 (52.2)	151 (47.8)	0.007 *	182 (57.6)	134 (42.4)	<0.001 *	123 (38.9)	193 (61.1)	0.107	97 (30.7)	219 (69.3)	0.976	161 (50.9)	155 (49.1)	0.094
	>21 years	120 (41.2)	171 (58.8)		124 (42.6)	167 (57.4)		95 (32.6)	196 (67.4)		89 (30.6)	202 (69.4)		168 (57.7)	123 (42.3)	
**Year of study**	1st year	57 (46.7)	65 (53.3)	0.122	64 (52.5)	58 (47.5)	0.187	43 (35.2)	79 (64.8)	0.537	38 (31.1)	84 (68.9)	0.018 *	68 (55.7)	54 (44.3)	0.248
	2nd year	64 (47.4)	71 (52.6)		75 (55.6)	60 (44.4)		56 (41.5)	79 (58.5)		31 (23.0)	104 (77.0)		65 (48.1)	70 (51.9)	
	3rd year	70 (55.6)	56 (44.4)		67 (53.2)	59 (46.8)		43 (34.1)	83 (65.9)		43 (34.1)	83 (65.9)		65 (51.6)	61 (48.4)	
	4th year	39 (37.9)	64 (62.1)		50 (48.5)	53 (51.5)		38 (36.9)	65 (63.1)		43 (41.7)	60 (58.3)		56 (54.4)	47 (45.6)	
	5th year	55 (45.5)	66 (54.5)		50 (41.3)	71 (58.7)		38 (31.4)	83 (68.6)		31 (25.6)	90 (74.4)		75 (62.0)	46 (38.0)	
**Marital status**	Single	246 (48.7)	259 (51.3)	0.053	264 (52.3)	241 (47.7)	0.041 *	185 (36.6)	320 (63.4)	0.411	147 (29.1)	358 (70.9)	0.068	271 (53.7)	234 (46.3)	0.554
	Married or cohabiting	39 (38.2)	63 (61.8)		42 (41.2)	60 (58.8)		33 (32.4)	69 (67.6)		39 (38.2)	63 (61.8)		58 (56.9)	44 (43.1)	
**Place of origin**	Capital	125 (39.6)	191 (60.4)	<0.001 *	138 (43.7)	178 (56.3)	0.001 *	107 (33.9)	209 (66.1)	0.272	84 (26.6)	232 (73.4)	0.024 *	159 (50.3)	157 (49.7)	0.045 *
	Province	160 (55.0)	131 (45.0)		168 (57.7)	123 (42.3)		111 (38.1)	180 (61.9)		102 (35.1)	189 (64.9)		170 (58.4)	121 (41.6)	
**Area of residence**	Urban	255 (46.8)	290 (53.2)	0.811	275 (50.5)	270 (49.5)	0.945	201 (36.9)	344 (63.1)	0.141	163 (29.9)	382 (70.1)	0.245	294 (53.9)	251 (46.1)	0.707
	Rural	30 (48.4)	32 (51.6)		31 (50.0)	31 (50.0)		17 (27.4)	45 (72.6)		23 (37.1)	39 (62.9)		35 (56.5)	27 (43.5)	
**History of mental illness**	Yes	25 (100.0)	0 (0.0)	<0.001 *	22 (88.0)	3 (12.0)	<0.001 *	25 (100.0)	0 (0.0)	<0.001 *	9 (36.0)	16 (64.0)	0.553	18 (72.0)	7 (28.0)	0.068
	No	260 (44.7)	322 (55.3)		284 (48.8)	298 (51.2)		193 (33.2)	389 (66.8)		177 (30.4)	405 (69.6)		311 (53.4)	271 (46.6)	
**Living with people vulnerable to COVID-19**	Yes	60 (47.2)	67 (52.8)	0.941	65 (51.2)	62 (48.8)	0.845	53 (41.7)	74 (58.3)	0.124	40 (31.5)	87 (68.5)	0.814	68 (53.5)	59 (46.5)	0.867
	No	225 (46.9)	255 (53.1)		241 (50.2)	239 (49.8)		165 (34.4)	315 (65.6)		146 (30.4)	334 (69.6)		261 (54.4)	219 (45.6)	

* Based on Pearson’s chi-square (*p* < 0.05, significant association).

**Table 3:** Adjusted regression analysis model of depression, anxiety, stress and fear of COVID-19 associated with the rate of temporomandibular disorders considering possible confounding variables.

Variable	Category	Crude Model					Adjusted Model				
		β	PR	95% CI		*p* *	β	APR	95% CI		*p* **
				LL	UL				LL	UL	
**Depression**	Yes	0.61	1.84	1.58	2.15	<0.001	0.32	1.38	1.15	1.66	0.001 **
	No		*Ref.*					*Ref.*			
**Anxiety**	Yes	0.73	2.07	1.75	2.45	<0.001	0.56	1.75	1.44	2.13	<0.001 **
	No		*Ref.*					*Ref.*			
**Stress**	Yes	0.45	1.57	1.37	1.80	<0.001	−0.03	0.97	0.83	1.12	0.657
	No		*Ref.*					*Ref.*			
**Fear of COVID-19**	Yes	0.25	1.28	1.11	1.48	0.001	0.01	1.01	0.88	1.16	0.857
	No		*Ref.*					*Ref.*			
**Sex**	Female	0.54	1.71	1.41	2.08	<0.001	0.44	1.55	1.28	1.87	<0.001 **
	Male		*Ref.*					*Ref.*			
**Age group**	≤ 21 years	−0.12	0.88	0.76	1.02	0.094	−0.14	0.87	0.73	1.05	0.139
	> 21 years		*Ref.*					*Ref.*			
**Year of study**	1st year	−0.11	0.90	0.73	1.11	0.324	−0.05	0.95	0.76	1.19	0.660
	2nd year	−0.25	0.78	0.62	0.97	0.027	−0.23	0.80	0.63	1.01	0.057
	3rd year	−0.18	0.83	0.67	1.04	0.101	−0.20	0.82	0.65	1.03	0.093
	4th year	−0.13	0.88	0.70	1.10	0.254	−0.15	0.86	0.70	1.06	0.168
	5th year		*Ref.*					*Ref.*			
**Marital status**	Single	−0.06	0.94	0.78	1.14	0.545	−0.04	0.96	0.80	1.17	0.713
	Married or cohabiting		*Ref.*					*Ref.*			
**Place of origin**	Capital	−0.15	0.86	0.74	1.00	0.045	−0.07	0.93	0.81	1.07	0.317
	Province		*Ref.*					*Ref.*			
**Area of residence**	Urban	−0.05	0.96	0.76	1.21	0.701	−0.04	0.96	0.77	1.20	0.708
	Rural		*Ref.*					*Ref.*			
**History of mental illness**	Yes	0.30	1.35	1.04	1.74	0.022	−0.15	0.86	0.68	1.10	0.228
	No		*Ref.*					*Ref.*			
**Living with people vulnerable to COVID-19**	Yes	−0.02	0.98	0.82	1.18	0.868	0.00	1.00	0.85	1.19	0.973
	No		*Ref.*					*Ref.*			
**Model constant**							−1.12	0.33	0.23	0.45	<0.001

* Crude simple regression model (* *p* < 0.05, significant association), PR: Prevalence Ratio. ** Adjusted multiple regression model (** *p* < 0.05, significant association), APR: Adjusted Prevalence Ratio under Poisson regression model with robust variance, β: Coefficient of determination. 95% CI: 95% Confidence Interval; LL: Lower Limit; UL: Upper Limit.

## Data Availability

The data presented in this study are available on request from the corresponding author.

## Abbreviations

APR: Adjusted Prevalence Ratio; CI: Confidence interval; COVID-19: Coronavirus disease 2019; DASS: Depression, Anxiety and Stress Scale; SARS-CoV-2: Severe Acute Respiratory Syndrome Coronavirus 2; SFAI: Short Form Anamnestic Index; TMD: Temporomandibular Disorders; SPSS: Statistical Package for the Social Sciences; STROBE: STrengthening the Reporting of OBservational Studies in Epidemiology.
